# Do We Look Like Our Siblings’ Names? A Socio-Onomastic Perspective on the Face–Name Matching Effect

**DOI:** 10.5334/irsp.1243

**Published:** 2026-04-14

**Authors:** Steven Verheyen, Jonathan Van den Berckt, Tom Heyman

**Affiliations:** 1 Erasmus University Rotterdam, Rotterdam, the Netherlands; 2 KU Leuven, Leuven, Belgium; 3 Leiden University, Leiden, the Netherlands

**Keywords:** face perception, naming, social influence, socio-onomastics, stereotypes

## Abstract

This Registered Report pertains to the face–name matching effect (Zwebner et al. 2017), according to which people can match the first name to an unknown target face above chance level. The purpose of the Registered Report was twofold: (i) to perform an independent conceptual replication of the face–name matching effect, and (ii) to establish the nature of the effect. To this end, undergraduate students were presented with uniform pictures of unfamiliar faces of similar age and ethnicity and asked to select these targets’ first names among two alternatives that were equated for length. Half of the trials constituted target trials, in which a face was accompanied by the true name of the depicted person and a filler name. The other half constituted sibling trials, in which a face was accompanied by the name of the same-sex sibling of the depicted person and a filler name. The target trials were meant to establish the generalizability of the face–name matching effect. If participants could reliably match a person’s true name to their face with these homogeneous materials, it would indicate that it is a pervasive effect that extends obvious ethnic, cultural, and zeitgeist differences. The sibling trials were meant to establish whether the effect extends to sibling names. If participants could also reliably match a face and the name of the depicted person’s sibling, it would indicate that we entertain stereotypes for types of names, of which individual names could be regarded as tokens. Such a finding would be in line with predictions from the field of socio-onomastics, according to which names are vehicles for social meaning creation that are systematically governed by the socioeconomic characteristics of the name givers. If the face–name matching effect for target trials were to exceed that of sibling trials, this would constitute evidence that names also carry additional, individuating information. Our results provided no conclusive evidence in support of either the face–name matching effect or an analogous effect involving sibling names. Although participants’ accuracy in identifying true names and sibling names slightly exceeded chance, these effects were not statistically significant, and Bayesian analyses yielded inconclusive evidence. Moreover, no significant difference was found between performance on target versus sibling trials, and Bayesian analysis provided evidence in favor of the null hypothesis of no difference. These findings indicate constraints on the generalizability of the face–name matching effect and suggest that its emergence depends on the heterogeneity of the stimulus materials.

According to the face–name matching effect, we look like our names. In a series of experiments, Zwebner et al. (2017) showed that participants who viewed pictures of people who were unknown to them performed above chance level when asked to identify the true name of the depicted persons from a list of names. The face–name matching effect occurs among pictures of individuals of the same sex and ethnicity, but is culture-dependent. It only shows when the depicted persons and the judges come from the same culture (Zwebner et al. 2017, Study 5). That is, the effect appears to rely on shared knowledge of name and face stereotypes.

The ability of social perceivers to match a face and a name is strongly driven by facial appearance regions that are under the depicted person’s control, such as one’s hairdo (Zwebner et al. 2017, Study 6 and Study 7). Zwebner and colleagues refer to given names as social tags that may have a Dorian Gray effect (Zebrowitz & Collins 1997) on one’s facial appearance.^1^ The argument goes that a name given to a child carries particular social expectations, which are realized in the child’s later appearance, much like cultural or social stereotypes may affect how we feel and act (Greenwald et al. 2002; Greenwald & Banaji 1995; Wheeler & Petty 2001). Name stereotypes are externalized through a self-fulfilling prophecy, as individuals adhere to social expectations of what a particularly named person should look like by wearing their hair or cosmetics “appropriately” (see also Naumann et al. 2009).

The face–name matching effect is supported by the observation that people appear to entertain facial stereotypes for first names. Lea et al. (2007) had one group of participants use face creation software to construct faces that matched particular names. They then created a face prototype for each name by averaging the created faces. These prototypes were then successfully matched to the target names by an independent group of participants, suggesting that people have expectations regarding the appearance of someone named Bob or Bill. The idea that people are able to extract social information from faces (McArthur & Baron 1983) dates back at least to Galton (1879), who purported that one could create ideal portraits of types of men, such as criminals, by superimposing pictures of individuals who could be considered tokens of the type. The face–name matching effect suggests that names too can be considered types, whose individual tokens (the name bearers) share stereotypical facial features.

Socio-onomastics provides information about the origin of name stereotypes. Socio-onomastics is the study of names in society and as such is concerned with social influences on naming practices (Ainiala 2016). It deals with the manner in which we use names to construct social identities and the related perception and evaluation of names (Ainiala & Östman 2017). In the field of socio-onomastics, it is clearly acknowledged that names are not just a means to identify or refer to individuals. The choice for a particular name often constitutes an act of identity creation by which parents express the heritage and intended future of a child. It has long been recognized that, as a result, names also serve the pragmatic purpose of social classification (Flugel 1930; Oberndorf 1920). The social meaning carried by names extends beyond such demographic classifications as gender and ethnicity, and includes socioeconomic status (SES) (Gaddis 2014; Joubert 1994), but also dimensions like intellect, warmth, and competence (e.g., Kasof, 1993; Newman et al., 2018). On this topic, Young et al. (1993, pp. 1770–1771) wrote:

“*It would seem that names differ from one another, and that each carries with it a list of characteristics and meanings that are unique to that particular name. Based on these data it would seem apparent that first names have differing characteristics and that people do respond to them differently*.”

The names we give our children are informed not only by our nationality, language, and religion, but also by our education, social values, and status (Ainiala & Östman 2017; Aldrin 2017). Investigations of name-giving motives have, for instance, revealed that many highly educated Europeans opt to give their children traditional names, while fashionable names are often preferred by less-educated people (e.g., Vandebosch 1998). Naming conventions like these explain why the co-occurrence of given names in families yields name clusters with different cultural, ethnic, or linguistic backgrounds (Bloothooft & Groot 2008). Although the relationship between name schemes and socioeconomic variables is quite complex (Bloothooft & Onland 2011), these observations nevertheless show that people of different backgrounds tend to choose different names for their children. The predominant use of certain (clusters of) names by people from different backgrounds is likely to be at the basis of name stereotypes. When traditional names are predominantly chosen by highly educated parents, traditional names might yield certain expectations like a higher than average intellect. To the extent that the same socioeconomic variables that give rise to these naming conventions are also exemplified in people’s appearance, they may provide the basis of the face–name matching effect. Rather than considering names types, of which individual name bearers are tokens, we might therefore consider names themselves tokens of a name type (i.e., the name clusters mentioned above) that signal certain socio-cultural expectations.

This raises the question of whether the face–name matching effect should be considered an individual name effect or a name type effect. When participants are able to match a name to a face, are they picking up on the externalization of expectations that can be expressed by a range of similar names or on name-specific expectations? We contend that sibling names allow one to investigate this. Children born in the same family share a myriad of socio-economic circumstances, including those that inform naming practices and expectations, yet carry different names. If the face–name matching effect reflects the externalization of individual name stereotypes, a person should look more like their own name than like their sibling’s name. If the effect reflects the self-fulfillment of name-type stereotypes, a person could equally look like their sibling’s name.

The purpose of the current study is two-fold. First, we want to conduct an independent conceptual replication of the face–name matching effect. Although Zwebner et al. (2017) show the effect in a series of studies and we probably all have had the anecdotal experience of encountering a person who looked exactly like her name (see, for instance, pop culture references such as “s/he is such a Karen/Chad”), the finding that we rather consistently pick an unknown person’s true name from a set of alternatives, remains surprising as most names tend to be shared by numerous different looking individuals. The amount of press coverage Zwebner et al.’s study garnered (including features in Harvard Business Review, The Huffington Post, and The Today Show) testifies to this. We feel it is important to establish the generalizability of the face–name matching effect. Depending on the situation, the ability to match a name to a face varies from the rather unimpressive (e.g., when the choice is between names that differ clearly in gender or ethnicity, and this information is also apparent in the picture) to quite remarkable (e.g., in which respects do Bill and Bob really differ?). A conceptual replication with tightly controlled stimulus material therefore seems warranted to establish any constraints on the generalizability of the effect. To our knowledge, such a replication has not yet been undertaken, although Kramer and Jones (2015) failed to find evidence for a name-face matching effect in two studies preceding Zwebner et al.’s publication, using a paradigm in which participants had to identify the picture that matched a target name (rather than the other way around, as in Zwebner et al.).

Second, we want to further investigate the nature of the (potential) face–name matching effect. According to Zwebner et al. (2017), self-fulfillment of the social expectations that come with a name is an important source of information for matching a face and a name. Individuals supposedly adjust the aspects of one’s facial appearance that are under one’s control to these social expectations. People who are aware of the prevailing social standards with respect to naming and appearance can then capitalize on this information to identify names and appearances that best go together. If this explanation for the face–name matching effect holds, we predict that participants should also be able to match a face to the name of that person’s same-sex sibling. From the socio-onomastic literature, we know that a family’s background is predictive of their offspring’s names (Ainiala & Östman 2017; Aldrin, 2017; Vandebosch 1998), resulting in clusters constituted of similar names (Bloothooft & Groot 2008). If we take the socio-onomastic idea of social identity creation through naming or “social tagging” to heart, the prototypes associated with siblings’ names should be similar, assuming that parents have similar expectations for their children.^2^^,^^3^ Put differently: Since they are based on similar considerations, the names of the children in a family will often share characteristics, which are likely to yield comparable attitudes and expectations. If the shaping of one’s appearance through self-fulfillment of such expectations is what is driving the face–name matching effect, we expect it to extend to sibling names. If there is more to the effect, in that the individual name adds to it, the face–name matching effect should be more pronounced for true names than for sibling names.

In what follows, we propose a study in which standardized pictures of individuals of comparable age and ethnicity are accompanied by names of similar length; either the name of the depicted individual and an alternative name, or the name of the depicted individual’s sibling and an alternative. The study offers an independent conceptual replication of the face–name matching effect in a different culture and language than the ones considered by Zwebner et al., with more homogeneous stimulus material. The proposed study also deviates from Zwebner et al. (2017) in that we will include two names per picture instead of four or five, and include more trials than they did (see *Material selection* for motivation). We would deem the face–name matching effect generalizable to these circumstances if participants would be better than chance at identifying the depicted persons’ names among the alternatives. This would indicate that the face–name matching effect is a pervasive effect that extends obvious ethnic, cultural, and zeitgeist differences. For the effect to extend beyond true names to sibling names, participants would also have to identify the names of the depicted persons’ siblings with an accuracy that is significantly higher than chance. This would locate the source of the face–name matching effect in expectations regarding a type of name, rather than in individual name stereotypes, which could then be regarded as tokens of a broader class or cluster of names with a particular sociological origin. Finally, if the face–name matching effect would be established both for true names and sibling names, but were to be more pronounced for true names, it would suggest that names carry additional, individuating information.

There is now ample empirical evidence that people are discriminated against based on their name in domains as diverse as education (Anderson-Clark, Green & Henley 2008; Erwin & Calev 1984; Harari & McDavid 1973), housing (Carpusor & Loges 2006; Feldman & Weseley 2013), and employment (e.g., Bertrand & Mullainathan 2004; Cotton, O’Niell & Griffin 2008; Moss-Racusin et al. 2012). Our given names also appear to influence people’s first impressions of us (Erwin 1995; Mehrabian 2001; Mehrabian 1997) and our social standing (Hartman, Nicolay & Hurley 1968; McDavid & Harari 1966). With the proposed study, we intend to acquire a better understanding of the way names acquire their stereotypical properties, which may eventually help counteract some of its negative influences.

## Material Selection

We composed a set of uniform pictures of individuals of the same age and ethnicity, together with their first name and the name of a same-sex sibling. We therefore introduced our research plan to two large groups of students (undergraduate history and criminology students) in introductory psychology courses at the University of Leuven (in Leuven, Flanders, Belgium) and asked them whether we could use their student ID picture and the name they go by.^4^ Male students were also asked to provide the name their brother (if any) goes by. Similarly, female students were asked to provide the name their sister (if any) goes by. In case students had more than one same-sex sibling, we asked them to provide the name of the same-sex sibling closest in age to them so as to increase the possibility that similar conventions and considerations determined the name choice. In the instructions, we made it clear that they should not convey the names of siblings who were adopted or part of a blended family, since the name choice considerations could have been different for them (Johnson et al. 1991). They were also asked to answer a number of demographic questions (age, nationality, home language) so that we could guarantee the uniformity of the stimuli (see below). Finally, students indicated the highest diploma obtained by their parents, so as to give us a rough indication of their social background. They received two euros in compensation for the use of their picture and information.

A total of 185 students agreed to the use of their student ID picture, of which 114 had a same-sex sibling and were therefore eligible for inclusion in our study. Thirteen pictures were not considered because the depicted students did not have the Belgian nationality and/or did not speak Dutch exclusively at home. This measure was taken to ensure that all included stimuli would be of the same ethnicity and therefore not suspect to different naming conventions and/or superficial recognition cues such as skin color. An additional eight pictures were not considered because their appearance differed markedly from the other pictures (different background, faces occupying a considerably larger or smaller area of the picture). This measure ensured a uniform set of pictures. One picture of a female student was deemed ineligible because her name was also typical for men. We decided to exclude this stimulus because bi-gender names might invoke different response behavior. Participants could have a preference to match non-bi-gender names to a face, for instance. To avoid name repetition in the experiment, we excluded pictures of people with the same name at random. After ensuring that every name only occurred once, we were left with 68 pictures (48 female, 20 male). The depicted students were all born in 1999, 2000, or 2001, and thus of similar age (estimated to be aged between 16 and 19 years old at university enrollment, the time the picture was taken). These students’ parents tended to be highly educated, with 75% of mothers and 60% of fathers having obtained a diploma beyond compulsory education. The corresponding 2*68 target and sibling names were subsequently used to form 34 quadruples, each comprised of two pairs of same-sex sibling names (i.e., 24 female quadruples comprised of 2 pairs of sisters and 10 male quadruples comprised of 2 pairs of brothers). The two pairs of siblings in a quadruple were matched as well as possible on the length of their names. For instance, if one pair of siblings had names with four and six letters, the other pair was also required to have names with (approximately) four and six letters. This measure was taken because there tends to be a strong correlation between the length of siblings’ names. It was not possible to equate the names on any other variables, such as the first letter or frequency because of the restricted stimulus set and the lack of publicly available name norming data in Flanders, Belgium. Table 1 lists all of the quadruples.

**Table 1:** Female and Male True Names with Same-Sex Sibling Names, Organized in Quadruples.


FEMALE NAMES				MALE NAMES			
	
TARGET	SIBLING	TARGET	SIBLING	TARGET	SIBLING	TARGET	SIBLING

Yinthe	Lincy	Louise	Emma	Charles	Henri-Felix	Michiel	Matthias

Cato	Lotte	Iris	Joyce	Ralph	Bernd	Kevin	Joost

Frauke	Lobke	Celien	Hanne	Xander	Jelle	Thomas	Robbe

Axelle	Ellen	Dagmar	Lenie	Tijmen	Joppe	Vincent	Ignace

Lena	Anna	Saar	Kaat	Joris	Koen	Lode	Seppe

Janne	Reine	Maïté	Estée	Jarne	Tom	Brent	Tim

Jade	Nymee	Marte	Kato	Siebe	Epke	Mats	Wout

Lindsay	Morgane	Katelijn	Lauren	Sander	Ruben	Lukas	Rafael

Lien	Sara	Noor	Fien	Alexander	Daan	Pieter-Jan	Tijl

Suzanne	Jakoba	Evelien	Astrid	Louis	Wannes	Jonas	Viktor

Yasmine	Amber	Chloé	Aurélie				

Sylvie	Jolien	Karlijn	Mariet				

Floriane	Paulien	Maurissa	Malissa				

Lieselotte	Charlotte	Louise-Marie	Alexandra				

Zoë	Yani	Caro	Bo				

Silke	Saartje	Laure	Carolien				

Sanne	Eva	Jana	Innez				

Julie	Elise	Elona	Hanna				

Chloë	Christina	Ann-Sophie	Nieke				

Joni	Maxine	Mirthe	Ilke				

Eline	Nathalie	Annelien	Sofie				

Delphine	Laura	Madelein	Marta				

Imke	Tille	Titia	Lisa				

Noa	Giulia	Ine	Jolande				


## Method

We report how we determined our sample size, all data exclusions, all manipulations, and all measures in the study (Simmons, Nelson & Simonsohn 2012). The proposed procedure was approved by the KU Leuven Social and Societal Ethics Committee (reference number G - 2019 03 1574). The study’s methodology, including the analysis code, was preregistered before collecting any data. After initiating the preregistration, we made three changes to the code: we included participant information (i.e., age and gender distribution), added confidence intervals for all parameter estimates, and fixed a mistake when importing the stimulus information file (i.e., the first row was incorrectly considered to contain variable names). In addition, the code for the Supplementary Materials and the by-item tests as such were not preregistered. Finally, we corrected a typo in the stimulus information file (i.e., the name Esteé should have read Estée). Importantly, in the experiment itself, all names were shown as intended. Other than that, there were no deviations from the preregistration.

### Participants

We were interested in testing whether the face–name matching effect observed by Zwebner et al. (2017) replicates in more homogeneous circumstances and whether it extends to sibling names. Presuming it exists, the latter effect will be of the same size or smaller than the actual face–name matching effect. Out of all the experiments in Zwebner et al., there is one condition that could inform us about the size of a potential sibling name effect. That is, in Study 6, Zwebner et al. found that participants could match people’s names to their respective hairstyles above chance. Given its modifiability, hairstyle is a very context-dependent facial feature reflecting social, cultural, and environmental factors. Because such factors are typically shared between siblings, we took the hairstyle effect as a proxy for a (potential) sibling effect. However, because it might have been an overestimate, either due to the sibling effect being smaller in reality, or because publication bias in general yields overblown effect sizes, we divided the reported effect size by two. This specific correction factor was based on the observation that effect sizes in replication studies are on average about half of the estimate reported in the original paper (Open Science Collaboration 2015).

In this specific case, we used the effect size on the logit scale from the mixed-effects regression analysis performed by Zwebner et al., as we will carry out the same analysis in the current paper (but with a chance level of 50% rather than 25%, see below). This yielded an effect size of 0.36/2 = 0.18, which was then used to determine the sample size using the simr package (Green & MacLeod 2016). The number of items depended on availability (i.e., 68 (see section *Material selection*)). The number of participants required to achieve a power level of 90%, assuming a true effect size of 0.18, is 80 (based on 200 simulated datasets per combination).

Participants were recruited among the University of Leuven first-year psychology students and were compensated with course credit. To minimize potential acquaintance with the students depicted in the pictures, the study was conducted in another academic year than the one in which the pictures were obtained. Participants also indicated whether they felt they recognized any of the persons in the pictures (see below).

### Materials

All materials were presented in Dutch, the official language of Flanders (Belgium), where the study was conducted. The experiment was implemented in Qualtrics (Qualtrics, Provo, UT).

**Figure 1:**
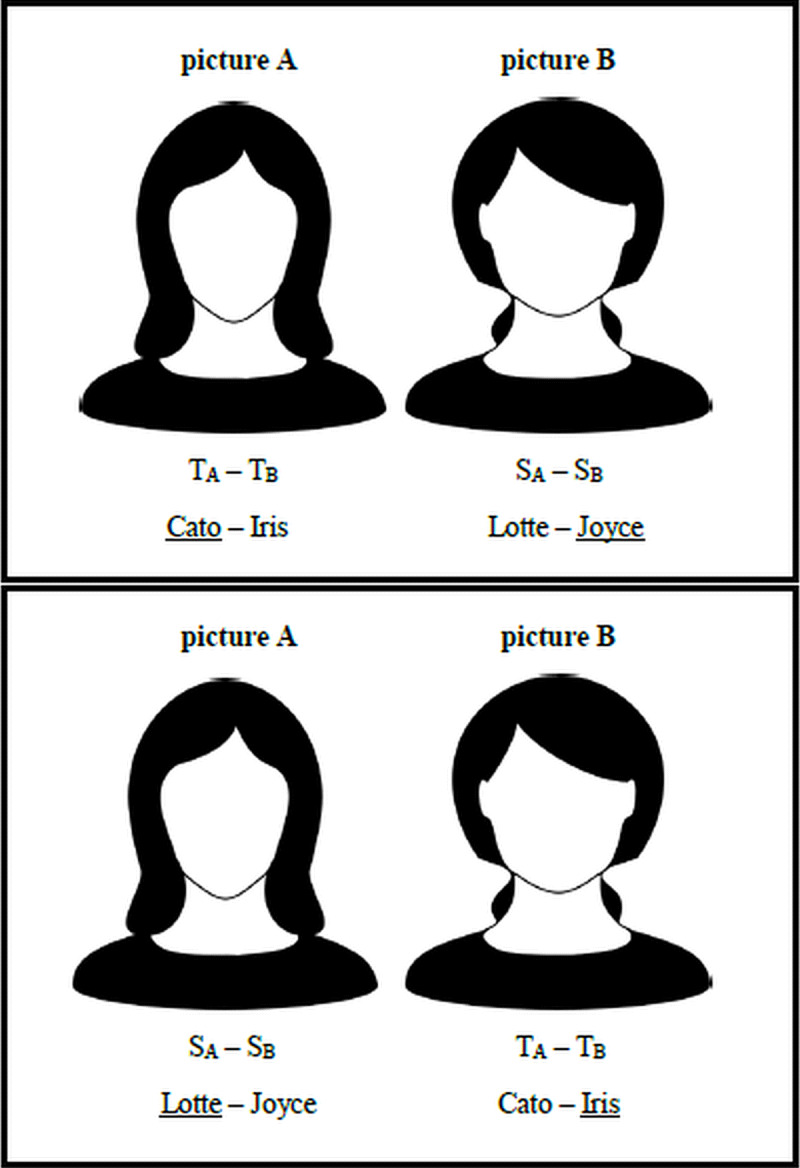
Schematic illustration of target and sibling trials. Picture A represents a woman whose true name (*T_A_*) is Cato and who has a sister called Lotte (*S_A_*). Picture B represents a woman whose true name (*T_B_*) is Iris and who has a sister called Joyce (*S_B_*). Half of the participants see picture A, accompanied by the target names, and picture B accompanied by the sibling names (top). The other half of the participants see picture A, accompanied by the sibling names, and picture B, accompanied by the target names (bottom). The answers that constitute evidence for the face–name matching effect are underlined, but this was not the case in the actual experiment. In the experiment, the presentation order of pictures and names was randomized (see section *Procedure*).

Each experimental trial consisted of a 300 by 400 pixels front-facing student ID picture, taken against a uniform light gray background from a similar distance. Each picture depicted a Flemish female or male student, aged between 16 and 19 years old, who has a same-sex sibling (see section *Material selection* on how these pictures were obtained). The students’ faces took up most of the pictures. All pictures showed the students’ hair, and some pictures revealed parts of their clothing. One could argue that these pictures somewhat increase the odds of finding the face–name matching effect in that facial expression, hair, cosmetics, jewelry, and clothing were under the depicted individuals’ control. That is, in their student ID, they could present to the world the face they wanted, adding to the ecological validity of the study. From the 24 female and 10 male quadruples, we constructed 68 experimental trials. Half of the participants saw one picture of a quadruple (picture A), accompanied by the true name of the depicted person (T_A_) and the true name of the person depicted in the other picture (picture B) of the quadruple (T_B_). We will refer to these trials as target trials. For these participants, picture B was accompanied by the sibling names of the persons depicted in the two pictures of that quadruple (S_A_ and S_B_). We will refer to these trials as sibling trials. The pictures–names combination was reversed for the other half of the participants. For them, the sibling names (S_A_ and S_B_) accompanied picture A (rendering it a sibling trial), while the true names (T_A_ and T_B_) accompanied picture B (rendering it a target trial). This way, a picture was always accompanied by two gender-appropriate names of comparable length (two male names in case a male was depicted; two female names in case a female was depicted), and no picture or name was presented more than once. Figure 1 illustrates the procedure schematically using four actual names from Table 1. In addition to the experimental trials, we also included an example trial, two practice trials, and two control trials. The example and practice trials provided participants the opportunity to experience the procedure they needed to follow during the experimental trials and ask questions to the experimenter in case anything was unclear. The control trials were intended to check whether participants were aware of the prevailing name conventions. The first control trial consisted of a picture of a prototypical female (a young lady with long blond hair), and the second control trial depicted a prototypical male (a young gentleman with apparent facial hair). Both pictures were drawn from the set of volunteered pictures by individuals without same-sex siblings and were similar in appearance to the experimental stimuli. The female picture was accompanied by the names Olivia and Arthur, the most popular girls’ and boys’ names in Flanders in 2019 (Flemish government, n.d.). The male picture was accompanied by the names Ella and Liam, the fifth most popular girls’ name and the second most popular boys’ name in Flanders in 2019 (the second, third, and fourth most popular girl names were already among the experimental stimuli).

### Procedure

Participants were tested individually and received a Dutch translation of the instructions used by Zwebner et al. (2017, p. 532):

*This research examines impression formation regarding people. Many times in life, we meet a person for the first time or only see his or her picture, and need to make a few decisions without any additional information. This study tests situations of this kind, and its goal is to examine the first impression people have of other individuals according to their facial appearance. Therefore, there is no right or wrong answer but only your “gut feeling”. Next, photos of individuals will appear. Your task now is to determine what is the true given name of each one of them*.

All participants started by viewing the same example, performed two practice trials, and were provided with the opportunity to ask questions. They were then presented with the 68 experimental trials in a random order. Each trial was presented on a separate screen. Half of the trials constituted target trials, in which a picture was accompanied by the true name of the depicted person and the true name of a person of the same sex in another picture with which the first picture was paired. The other half constituted sibling trials, in which a picture was accompanied by the name of the sibling of the depicted person and the name of the same-sex sibling of a person of the same sex in another picture with which the first picture was paired (see Figure 1). Names appeared next to each other below the pictures and the order of the names was randomized. Above each picture, participants read: *“Which of the names below is the true given name of the person in the picture?”*. Participants were provided with the opportunity to take a short break after the 20th and the 44th experimental trials in order to alleviate concerns about fatigue.^5^

After completing the experimental trials, participants were presented with the two control trials to check whether they were paying attention. As was the case for the experimental trials, the control trials were shown on separate screens in a random order. The names accompanying the pictures were shown in a random order as well. Participants were given the same instructions as in the experimental trials. We assumed that participants who were paying attention and were aware of the prevailing naming conventions will have made gender-appropriate choices on the control trials.

After completing the control trials, participants were asked whether they knew any of the people in the pictures personally. If they responded positively to this question, they were presented with all 70 pictures (experimental + control) they saw during the experiment, randomly organized in an 18 by 4 grid, and asked to indicate any person they knew.

Finally, participants were invited to indicate their gender (male/female/I do not identify with one of these categories), age (in years), nationality (Belgian/Other), and home language (Dutch/Other). After completing the demographic questions, participants were thanked for their participation and debriefed.

## Analysis

### Data exclusion

We only considered data from participants who had the Belgian nationality, spoke Dutch at home, were not older than 19 years, completed the entire study, passed the two control trials, and did not know any of the people in the pictures personally.

Note that these requirements are similar to the ones we applied to the selection of the stimulus materials. These exclusion criteria ensured that participants were familiar with the naming practices that governed the names that constituted our stimuli. Having the Belgian nationality and having Dutch as the home language ensured that all participants grew up in Flanders, the northern community of Belgium, where the study was conducted. This is important because naming practices vary regionally and between ethnicities (e.g., Bloothooft 2002; Lieberson & Bell 1992; Vandebosch 1998) and the face–name matching effect was found to be culture-dependent by Zwebner et al. (2017).

We required participants to be of a similar age as the individuals depicted in the pictures, since naming practices change with time (e.g., Barucca et al. 2015; Bush, Powell-Smith & Freeman 2018; Gallagher & Chen 2008) and we suspected that the responses by similarly-aged participants were only governed by the conventions that applied to the naming of their own generation, while the responses by participants who were significantly older than the depicted individuals may potentially have resulted from a mix of previous naming conventions (Kramer & Jones 2015). We also believe this choice adds practical relevance to the study in that investigating potential biases in impression formation seems most pertinent in the context of the relationships participants are most likely to engage in (i.e., relationships with peers).

The requirement to complete the entire study follows from the fact that the demographic information required to determine participant eligibility was only gathered at the end of the experiment (see *Procedure* section). Participants needed to answer both control trials with the gender-appropriate name (see *Materials* section) to demonstrate that they were taking the study to heart rather than answering carelessly, and were aware of the prevailing naming conventions. The data from participants who indicated to be familiar with any of the people in the pictures were excluded because it might have made them become aware of the experimental manipulation or confused them (i.e., in the case of sibling trials).

Participants who did not meet the above criteria were replaced so as to attain the desired number of 80 participants. The eventual sample size indeed corresponded with the desired number (*N* = 80) of which 16 were male and 64 were female, with a mean age of 18.24 (*SD* = 0.43).

### Statistical analyses

To assess whether the face–name matching effect replicates, we first performed a mixed-effects logistic regression analysis using only participants’ responses to the target trials (see Figure 1). That is, we fit a model with a general intercept, as well as by-participant and by-picture random intercepts, to the trial-level accuracy data (0 indicating incorrect, 1 indicating correct).^6^ As this involves a transformation into log-odds, chance-level performance (i.e., 50%) entails that the intercept is equal to zero. Hence, we test whether the intercept is significantly greater than zero, which would be expected if there is a face–name matching effect (unless we make a Type-2 error). To this end, we used the glmer function from the lme4 R package (Bates et al. 2015).

Furthermore, we also carried out an analogous Bayesian model comparison with the brm and bayes_factor functions from the brms R package (Bürkner 2017; Bürkner 2018). As a prior for the intercept, we used a positive half-normal with a standard deviation of 0.22, such that the mean of the distribution corresponds with the effect size estimate used for the power calculation (see above). This entails that considerable weight is placed on rather small effect sizes. For the other parameters, we used the default settings. Four chains were run with 10,000 iterations per chain, though the first half was discarded each time (known as burn-in). In addition, we conducted the exact same set of analyses for the sibling trials to test whether someone’s face might also match above chance with their sibling’s name. For comparability with Zwebner et al., we also report the outcomes of t-tests comparing participants’ accuracy proportion against chance in the Supplementary Materials.

Finally, we evaluated whether performance was better in the target condition than in the sibling condition. We therefore examined all data and added a dummy-coded condition variable (0 indicating a sibling trial, 1 indicating a target trial) as a fixed effect to the models specified above.^7^ In the frequentist analysis, we tested whether the effect is significantly greater than zero. For the Bayesian model comparison, we also defined a prior for this parameter. Again, we used a positive half-normal with a standard deviation of 0.22, so the mean of the distribution corresponds with the (conservative) effect size estimate used earlier. This may seem at odds with the priors proposed in the previous analyses, which placed identical weights on the parameter values in both conditions. The rationale is that we want to compare the results from those two analyses with one another, which would be complicated had we used different priors. The prior of the last analysis reflects the assumption that the effect size in the target condition is comparable to the result observed by Zwebner and colleagues (2017) in the hairstyle condition of Study 6. For the frequentist analyses, we use α = .05 (one-tailed) to establish significance. For the Bayesian analyses, we report Bayes Factors comparing the evidence for the competing hypotheses. Bayes factors above 3 or below 1/3 are considered as substantial evidence (Jeffreys 1961).

## Results

The analyses regarding the face–name matching effect on target trials only produced the following results. Frequentist analyses showed a non-significant effect: *b* = 0.09, 95% CI = [–0.07, 0.25], *z* = 1.09, *p* = .137. The point estimate translates to a probability to respond correctly of .52, where .50 is chance level. Bayesian analyses revealed that the data are slightly more in line with the null hypothesis compared to the alternative, but the Bayes factor did not reach the preregistered substantial evidence threshold of 1/3 (or 3): *BF*_10_ = 0.95.^8^ The results from both analyses do not unequivocally lead to a particular conclusion, hence the status of the face–name matching effect remains unclear based on these data, at least under the current circumstances (i.e., stimulus selection, procedure, and so on).

The analyses regarding the face–name matching effect on sibling trials only produced the following results. Frequentist analyses showed a non-significant effect: *b* = 0.10, 95% CI = [–0.10, 0.30], *z* = 0.98, *p* = .163. The point estimate translates to a probability to respond correctly of .52, where .50 is chance level. Bayesian analyses revealed that the data are slightly more in line with the null hypothesis compared to the alternative, but the Bayes factor did not reach the preregistered substantial evidence threshold of 1/3 (or 3): *BF*_10_ = 0.99. The results from both analyses do not unequivocally lead to a particular conclusion, hence the status of the sibling name effect remains unclear based on these data, at least under the current circumstances (i.e., stimulus selection, procedure, and so on).

Finally, the analyses comparing the magnitude of the difference between target and sibling trials produced the following results. The frequentist analysis showed a non-significant effect, *b* = –0.01, 95% CI = [–0.12, 0.10], *z* = –0.24, *p* = .596, and according to the Bayesian analysis, the data were more in line with the null model relative to the alternative model, *BF*_10_ = 0.21. That said, the results for the target and sibling trials as such provide inconclusive evidence regarding the existence of a face–(sibling)name matching effect, so the notion that these two effects are not considerably different from each other may not come as a surprise. The *t*-tests (see Supplementary Materials) similarly reveal no substantial evidence for a face–(sibling)name matching effect that generalizes across contexts.

It may be relevant to note that upon performing the frequentist analyses, all model fits returned singularity warnings. Upon closer inspection, this was caused by the random participant intercepts showing (practically) no variability. The latter was confirmed in the Bayesian analyses, where the estimates of the corresponding variance component were close to zero. In other words, these results suggest that participants performed quite similarly on the task. Note that removing the random effect from the model did not have a substantial impact on the outcomes of the frequentist analyses.

## General Discussion

The face–name matching effect refers to the phenomenon where people can match a person’s face to their true first name at above-chance levels (Zwebner et al. 2017). In this Registered Report, we set out to replicate the effect under tightly controlled conditions and to test whether it extends to the names of same-sex siblings. Our results provide no conclusive evidence for a face–name matching effect or an extension of the effect to sibling names. Bayesian and frequentist analyses alike indicated that participants’ performance was close to chance across conditions. We interpret these results to mean that the face–name matching effect is context-dependent. Whereas earlier demonstrations of the effect used more diverse stimuli, our study controlled for ethnicity, age, and name length. The majority of the depicted individuals also grew up in similar, higher socioeconomic strata. This homogeneity may have minimized the usefulness of extraneous cues that could otherwise guide name decisions. The absence of strong effects in the current data indicates constraints on the generalizability of the face–name matching effect.

To our knowledge, no independent replication of the face–name matching effect has been undertaken, although the authors of the original paper have presented a follow-up in which they successfully replicate the effect with both adult and child participants (Zwebner et al. 2024). Our study involved a conceptual replication with tightly controlled stimulus materials. On target trials, unfamiliar faces were accompanied by the true name of the depicted person and one filler name, instead of three or four as in Zwebner et al. (2017). We used standardized pictures of individuals of comparable age (16–19 years of age) and ethnicity, which were accompanied by names of similar length, whereas the face and name stimuli in the original studies were more heterogeneous. In Zwebner et al. (2017), the average age of the target faces ranged from 20 to 30 years. Both of our studies involved student participants, although in terms of age, our participants were less diverse than in the original because they were deliberately chosen to have a similar age as the students depicted in the target pictures.

We considered the 2AFC version of the procedure proposed in Zwebner et al. (2017) as a way to study the face–name matching effect with better-matched controls. We saw no theoretical reasons why the face–name matching effect would not be present when participants are offered two instead of four names to match a face to. As far as we can tell, there is nothing in the original account of the effect that would suggest it is dependent on the number of alternatives. Based on our findings, we cannot rule out, however, that the decision process may proceed differently for a varied number of alternatives. In this regard, it also needs to be acknowledged that Kramer and Jones (2015), who failed to find evidence for a name-face matching effect, also presented two stimuli (in their study: faces) for participants to choose from.

Based on a yet unpublished replication that we conducted ourselves (Van den Berckt 2023) and that in terms of design is closer that of Zwebner et al. (2017) with four alternative names instead of two, we are confident ruling out the number of alternatives as a potential explanation, seeing that this study too failed to provide convincing evidence for the face–name matching effect. It yielded mixed results, with some trials (faces) demonstrating the face–name matching effect and others not. It also included a conceptual replication of Kramer and Jones (2015), where participants had to choose the correct face among four options to match a given name. The replication did not provide convincing evidence for a name-face matching effect either, in line with the original results. This suggests that the design or number of alternatives isn’t a decisive factor. Both replications did use homogeneous materials that are comparable to the ones in the current study, making this the most likely explanation for the diverging results.

We selected our materials (pictures and names) from students of the same university, who had grown up in largely similar socioeconomic circumstances and were enrolled in only two programs. They could potentially be argued to share similar parental expectations and social interactions, making for a homogeneous stimulus set if the social psychological (Greenwald et al. 2002; Greenwald & Banaji 1995; Naumann et al. 2009; Wheeler & Petty 2001; Zwebner et al. 2024; Zwebner et al. 2017) and/or socio-onomastic (Ainiala 2016; Ainiala & Östman 2017) considerations underlying the face–name effect were to hold. There is also the possibility that people do not look like their names by age 16–19. Zwebner et al. (2024) have shown that the face–name matching effect develops over time. They found that children learn name stereotypes early on, but do not yet show them in their appearance. That is, both adults and children were able to identify the names of unfamiliar faces of adults above chance, but not of 9-year-old children. It is possible that the externalization of the name stereotypes that Zwebner et al. (2017, 2024) propose was not yet complete in the depicted individuals in our study, thus attenuating the face–name matching effect. Although we cannot formally rule out the possibility that the face–name matching effect is smaller (under these circumstances) than we had assumed and that our results represent a false negative, it is important to remind the reader that we did start from a conservative effect size estimate that involved half of the originally reported effect.

On the other hand, the facial expression, hairstyle, cosmetics, jewelry, and clothing in our stimuli were under the depicted individuals’ control, and one could argue that they present an age group that is particularly sensitive to how they present themselves to the outside world. These are factors that could potentially increase the likelihood of observing the face–name matching effect, as in their student ID pictures, these young people were able to present themselves in a way that aligned with their intended self-image.^9^ Finally, since the age of the participants and the depicted individuals was closely matched, one could expect increased performance under these conditions because people exhibit a recognition bias toward people their own age due to increased contact and familiarity, and more in-depth processing of the faces of the members of one’s in-group (Brouwer et al. 2021). Yet, no compelling evidence for the face–name matching effect emerged.

Another difference with the procedure used by Zwebner et al. (2017) is that, in our study, the filler name for a given picture was a target (or sibling) name for a different picture. Participants never saw the same name or picture more than once, but it was balanced out across participants such that potential base rate preferences for a certain name would not artificially inflate or even “create” a face–name matching effect. In their Study 3, Zwebner et al. (2017) attempted to rule out the influence of name characteristics by demonstrating that the face–name matching effect did not occur when face information was absent (i.e., when participants were shown a black square and were forced to choose a name for the face that was supposedly hidden behind the square). However, we deem that control condition insufficiently natural to rule out these influences. It is not at all clear how participants would react under such circumstances and on which considerations they would base their answers. Zwebner et al. did conduct one experiment (i.e., Study 1B) in which filler names were sampled from the pool of targets, but, contrary to our study, participants saw names more than once. Consequently, this repetitive exposure raises concerns about nonindependence, as acknowledged by Zwebner and colleagues, and perhaps participants could notice a pattern. Taken together, it is possible that the current study yielded inconclusive evidence because we controlled for potential base rate name preferences in a stricter manner. This, again, raises concerns about the generalizability and size of the face–name matching effect.

Another interesting observation that emerged from the analyses was the lack of variability among participants. This showed both in the lack of variability among the participant intercepts in the frequentist mixed-model analysis and in the very small variance component in the Bayesian analysis. It thus doesn’t seem to be the case that there are individuals in our sample who are very skilled at accurately matching faces and names, whose performance is offset against that of people who are particularly bad at it. Our participants performed close to chance overall. The random stimulus effects were more pronounced, suggesting that the effect may differ from trial to trial. This indicates that the make-up of each trial should be carefully investigated in future work. Both the faces and the names can vary along several dimensions, such as the relative frequencies of the names and the similarity of the alternative names, that have not yet been accounted for in studies on the face–name matching effect. These dimensions of variation could interact to see the face–name matching effect present to different degrees, but could also have independent effects. The random stimulus effects we identified in our study could, for instance, indicate base rate preferences for certain names, regardless of the face they are accompanied with. Investigating this more systematically probably requires scaling up the research to include more varied stimuli and participants (e.g., Tkachenko & Jedidi, 2023) and exploiting the many norming data sets that are available for names (e.g., Joosse, Kuscu & Cassani 2025; Newman et al., 2018). More generally stated, it is reasonable to expect different faces to display name stereotypes to different degrees (Adams & Verheyen 2025), and different people to entertain name stereotypes to different degrees, although we found no evidence for the latter in our homogeneous participant sample.

Through this Registered Report, we had also hoped to gain further insight into the nature of the face–name matching effect. To this end, we included sibling trials, in which unfamiliar faces were accompanied by the name of the same-sex sibling of the depicted person and a filler name. The idea here was to test whether the face–name matching effect is driven by broader name-type stereotypes or by information unique to individual names. If participants could accurately match a face to the name of that person’s sibling, this would suggest that shared social stereotypes tied to name types guide such judgments—an idea rooted in socio-onomastic theory, which views names as carriers of socially constructed meaning shaped by the socioeconomic background of the name givers. If participants were to perform better on target trials (where a face is accompanied by the true name of the depicted individual) than on sibling trials, it would imply that names convey not only general social cues but also individuating information specific to the name bearer.

When participants are able to match a name to a face, are they picking up on the externalization of expectations that can be expressed by a range of similar names or on name-specific expectations? We found no difference between target and sibling trials. The lack of a stronger effect for true names compared to sibling names suggests that individual name stereotypes may not play a substantial additional role beyond broader expectations based on similar names that reflect comparable social expectations, at least in our homogeneous stimulus and participant sample. One potential interpretation of our findings is that the names we used all come with comparable expectations and are therefore difficult to tell apart because they all pertain to students with a comparable and relatively high SES. Future work could perhaps take up this comparison in more heterogeneous situations or by comparing true and sibling names directly with respect to the same picture. The strongest comparison would probably involve twins. If people are able to match each twin to their true given name better than to the other twin’s name, that would be very strong support for individuating information. Note though that such comparisons operate under the assumption that there is a reliable face–name matching effect to begin with. As we did not obtain substantial evidence for such an effect in the current study, research regarding the nature of the effect is arguably premature.

In summary, we haven’t been able to gain convincing answers as to the origin of the face–name matching effect and more specifically to the question whether names carry individuating information that extends beyond that shared by the names of their siblings, who arguably were chosen based on similar considerations. What our study did show is that there are constraints on the generalizability of the face–name matching effect. With more homogeneous stimulus material and potentially more homogeneous participants than in the study in which the effect was originally established (Zwebner et al. 2017), we were unable to provide convincing evidence for or against the effect, indicating that it does not necessarily extend across various procedural differences. Although this might come across as a rather modest contribution, we believe it is an important qualification that was omitted in the popular press coverage of the original result, in which people’s ability to guess a person’s name based on their appearance was heralded (see Broersma 2010, for the potential consequences of such an uncritical approach). Our results underscore the need for further research to delineate the boundary conditions under which the face–name matching effect may (or may not) emerge. It is our opinion that one could get any finding in between nearly perfect ability to match a face to a name, and no ability at all, simply by changing the homogeneity of the stimulus set and/or the nature of the rater population. In the more systematic exploration of these circumstances probably lies the answer to the nature of the face–name matching effect.

## Supplementary Materials

We performed a number of *t*-tests comparing average accuracy against chance (of which the code was not preregistered). First, we examined every participant’s performance on target and sibling trials separately. For both types of trials, average accuracy was significantly above 50%: *M* = 0.52, 95% CI [0.50,0.54], *t*(79) = 2.38, *p* = .020, for target trials, and *M* = 0.52, 95% CI [0.51,0.54], *t*(79) = 2.67, *p* = .009, for sibling trials. Second, we considered the performance on each picture across participants. Note that such a *t*-test was not reported in Zwebner et al. (2017), but we deem it important given that one not only wants to generalize across participants, but also across stimuli (see Clark, 1973). For both types of trials, average accuracy was not significantly above 50%: *M* = 0.52, 95% CI [0.48,0.56], *t*(67) = 1.11, *p* = .272, for target trials, and *M* = 0.52, 95% CI [0.48,0.57], *t*(67) = 1.06, *p* = .294, for sibling trials. Finally, we also looked at the performance on each name pair across participants. For both target and sibling name pairs, average accuracy was not significantly above 50%: *M* = 0.52, 95% CI [0.49,0.55], *t*(33) = 1.32, *p* = .197, for target name pairs, and *M* = 0.52, 95% CI [0.49,0.56], *t*(33) = 1.43, *p* = .161, for sibling name pairs. In conclusion, even though the by-participant analyses showed a statistically significant effect for both target and sibling trials, the two sets of by-item analyses suggest that the effect may be specific to certain items. These results align with the mixed-effects analyses in that there does not appear to be substantial evidence for a face–(sibling)name matching effect that generalizes across contexts.

## Open practices

The manuscript was written in R (R Core Team, 2016) using the packages papaja (Aust & Barth 2017) and rmarkdown (Allaire et al. 2016). It is publicly available as a Code Ocean container at https://doi.org/10.24433/CO.9972623.v4. The Stage 1 version of this Registered Report can be found on the Open Science Framework: https://osf.io/nygpu. The pictures that were used as stimuli are not made available to ensure the anonymity of the depicted individuals who volunteered the use of their student ID picture.

## Footnotes

In Oscar Wilde’s 1891 novel “The Picture of Dorian Gray,” the main character’s portrait changes as he commits various amoral acts.

This holds especially at the time the name is chosen, which often occurs prior to birth, when little is known about the child, and for children of the same sex who do not differ with respect to gender stereotypes. Admittedly, it is of course possible that parents’ expectations change as their family grows and/or that parents reserve the name that best communicates their expectancies for their first born, and that later siblings’ names communicate these expectancies less effectively.

Another possibility is that socioeconomic circumstances change. One sibling could be born when the family was in a different SES than when the other was born. We thank a reviewer for raising this point, which prompted us to add this footnote at Stage 2 of the RR procedure.

Note that this doesn’t necessarily have to be their given name, but could potentially be a nickname or shortened name. People who go by a nickname do not look like their given name (Zwebner et al. 2017, Study 8). Since we did not obtain this information, we do not know which names constitute given names and which ones constitute nicknames or shortened versions. It may thus not be possible to distinguish between people changing their visual presentation to fit their name and changing their preferred name version to fit their visual presentation.

For comparison, note that in Zwebner et al. (2017), participants matched between 10 and 25 faces to four or five name alternatives.

In terms of R syntax, the model looks as follows: correct ~ 1 + (1|pictureID) + (1|participantID). Note that in the code we use ppID to abbreviate the latter.

In terms of R syntax, the model looks as follows: correct ~ 1 + condition + (1|pictureID) + (1|participantID). As the current model already returned singularity warnings for the frequentist analyses, we decided not to fit more complex models, including random slopes (we revisit this in the Results section).

No Bayesian parameter estimates are reported because the prior distribution for the regression coefficient was geared toward hypothesis testing and as such placed no weight on negative values.

Although one might argue that at this age, they might also have wanted to present an image through which they distance themselves from their parents’ expectations.
